# Influence of Sorbitol and Glycerol on Physical and Tensile Properties of Biodegradable–Edible Film From Snakehead Gelatin and *κ*-Carrageenan

**DOI:** 10.1155/ijfo/7568352

**Published:** 2025-01-15

**Authors:** Rosmawati Rosmawati, Sri Fatmah Sari, Asnani Asnani, Wa Embe, Asjun Asjun, Dwiprayogo Wibowo, Irwan Irwan, Nurul Huda, Muhammad Nurdin, Akrajas Ali Umar

**Affiliations:** ^1^Department of Fishery Products Technology, Faculty of Fisheries and Marine Sciences, Universitas Muhammadiyah Kendari, Kendari, Southeast Sulawesi, Indonesia; ^2^Department of Fishery Products Technology, Faculty of Fisheries and Marine Sciences, Universitas Halu Oleo, Kendari, Southeast Sulawesi, Indonesia; ^3^Agribusiness Field Laboratory, Faculty of Agriculture, Universitas Muhammadiyah Kendari, Kendari, Southeast Sulawesi, Indonesia; ^4^Department of Fishery Products Technology, Institut Teknologi dan Bisnis Nobel Indonesia, Makassar, South Sulawesi, Indonesia; ^5^Department of Environmental Engineering, Faculty of Engineering, Universitas Muhammadiyah Kendari, Kendari, Southeast Sulawesi, Indonesia; ^6^Department of Pharmacy, Faculty of Sciences and Technology, Institut Teknologi dan Kesehatan Avicenna, Kendari, Southeast Sulawesi, Indonesia; ^7^Faculty of Fisheries and Marine Science, Universitas Brawijaya, Malang, East Java, Indonesia; ^8^Department of Chemistry, Faculty of Mathematics and Natural Sciences, Universitas Halu Oleo, Kendari, Southeast Sulawesi, Indonesia; ^9^Institute of Microengineering and Nanoelectronics, Universiti Kebangsaan Malaysia, Bangi, Malaysia

## Abstract

Two plasticizers with distinct properties are carefully studied in this research for their suitability in creating biocomposite edible film products. The study uncovers films' physical, tensile, and biodegradability attributes, using snakehead gelatin and ĸ-carrageenan in different concentrations, with sorbitol or glycerol as plasticizers. The biomaterials of the edible film consist of snakehead gelatin (*Channa striata*) 2% (*w*/*v*); ĸ-carrageenan at concentrations of 1%, 1.5%, and 2% (*w*/*v*); and sorbitol/glycerol 15% (*v*/*v*). The addition of ĸ-carrageenan up to 2% in the formulation increased the film thickness to 0.046 ± 0.005 mm, tensile strength to 2.05 ± 0.56 MPa, and elongation at break to 35.00% ± 2.92% while decreasing the water vapor transmission rate (WVTR) to 0.17 ± 0.00 g/mm^2^/h (*p* < 0.05). The effect of glycerol in the composite did not affect thickness and luminosity (*L*^∗^) (*p* > 0.05), but the tensile strength increased from 0.18 ± 0.17 to 1.03 ± 0.40 MPa (*p* > 0.05). Sorbitol increased the value of color difference, elongation at break, and WVTR, namely, 19.77 ± 1.02, 25.20% ± 1.79%, and 0.28 ± 0.02 g/mm^2^/h, respectively (*p* < 0.05). The swelling index of the films increased with ĸ-carrageenan concentration, whereas the water content decreased (*p* < 0.05). The addition of sorbitol reduced the solubility of the film from 71.43% ± 12.39% to 42.67% ± 15.44% (*p* < 0.05), while glycerol did not affect changes in film solubility (*p* > 0.05). The presence of sorbitol had no significant effect on the contact angle (*p* > 0.05) and was more affected by the addition of glycerol at higher ĸ-carrageenan concentrations (*p* < 0.05). The ability to decompose after 28 days was more remarkable for films containing glycerol than sorbitol. Fourier transform infrared analysis revealed the functional group structures of all samples, indicating that no new compounds were formed in them. The surface structure of the sorbitol-plasticized film was predicted to be rougher and easily cracked, but more compact and dense, while the glycerol-plasticized film tended to be smoother with fainter cracks due to its hygroscopic properties.

## 1. Introduction

Recently, there has been increasing awareness of transitioning from plastic packaging to edible packaging alternatives. This statement is a warning of the potential dangers of using plastic packaging due to its inherent challenges during the natural decomposition processes (characterized by delayed biodegradation) and its inability to be replenished or regenerated [1–3]. Due to these factors, there has been an increase in the investigation and development of natural packaging in response to issues related to health, consumption safety, renewable energy, and ease of degradation [4, 5]. Biopolymers such as proteins, polysaccharides, lipids, and their various combinations are good film biomaterials [2, 6], and the addition of plasticizers can improve the functional attributes of films and their characteristics [1, 7].

The type of protein that is widely applied and considered suitable for the packaging industry is fish gelatin. Fish gelatin is a by-product of processing in the fishing industry, which can change collagen into gelatin through a partial hydrolysis derivatization process [8]. One type of potential fish that has high economic value is snakehead fish. This fish is a source of albumin protein [9–11], and its by-products such as skin, bones, and scales are a source of collagen [12, 13]. Snakehead fish by-products have not been widely utilized, even though their potential as gelatin makes this by-product have utility value as a biopolymer. Chemically, snakehead gelatin (SG) has the potential to be applied as a biofilm, like fish gelatin in general, as proven by previous researchers [14, 15]. Rosmawati et al. [16] recommend the use of SG as a binder of approximately 2%. This information can be used as a reference for application purposes in producing various other SG–based products, including as edible biofilm materials. Fish gelatin has been widely studied as a promising film biomaterial. As a hydrocolloid protein macromolecule, gelatin can form a thin, translucent, and elastic solid layer [17]. Gelatin as a film base is intended to provide protection, preservation, or extension of the shelf life of food products [18], including protection against oxygen and light [19]. However, it should be considered that these films are also susceptible to the adverse effects of excessive humidity [20]. Gelatin has limited disintegration capacity, water solubility, and mechanical strength but exhibits biocompatibility, biodegradability, affordability, nontoxicity, and forceful hydrogen bonding [21] Thus, this protein-based film, which has forceful adhesive characteristics, can also effectively block oxygen and carbon dioxide permeation, although it is weak in preventing water diffusion [21, 22]. According to Ramos et al. [18], gelatin is usually the packaging material of choice for products susceptible to damage due to its unique functional features compared to other hydrocolloids.

Seaweed-based biomaterials, such as carrageenan (CAR), including nonstarch polysaccharide species from red algae, *Kappaphycus alvarezii* [23, 24], are hydrocolloids that are often used as a base material for biofilms. Although CAR shows the ability to decompose quickly, is nontoxic, has antioxidant properties, and readily forms a film layer [25, 26], like most polysaccharide biopolymers, CAR has poor water vapor barrier properties, is brittle, and is vulnerable to degradation [27]. The hydrophilic nature of CAR results in limitations in mechanical properties and weak permeability [28]. Several studies have been carried out to see the suitability and properties of films from combining CAR with other biomaterials, which can improve physical properties and mechanical characteristics and provide a good barrier [28, 29]. Farhan and Hani [1] have used 2% (*w*/*v*) CAR as an edible film material, and Rusli, Metusalach, and Tahir [30] recommended at most 3% (*w*/*v*), resulting in an edible film with better physical properties.

Sorbitol (SOR) and glycerol (GLY) are commonly used as plasticizers in producing edible films due to their ability to reduce the level of hydrogen bonding, thereby increasing the flexibility of the films [31]. According to Bakry, Isa, and Sarbon [32] and Fu et al. [33], GLY has limited tensile strength (TS) and shows increased moisture retention and absorption capacity; in contrast, SOR shows increased efficiency and efficacy when interacting synergistically with water. Plasticizers can improve mechanical strength, demonstrated good barrier properties, and can prevent brittleness due to high intermolecular forces [1]. For this reason, the novelty presented is to create a sustainable biopolymer from snakehead fish waste that produces gelatin combined with CAR as an environmentally friendly biopolymer material. The combination of SG and CAR needs to be tested to determine the suitability of the composite between the two biomaterials. This study is aimed at discovering edible films' physical, tensile, and biodegradability attributes based on SG and ĸ-carrageenan (ĸ-CAR) in different concentrations with SOR or GLY as plasticizers. Hence, this research's results hope to provide critical information for developing edible films made of SG and ĸ-CAR that are biodegradable, cheap, and applicable to future food packaging industries.

## 2. Materials and Methods

### 2.1. Materials

The scales and skin of snakehead fish were sourced from the conventional freshwater fish market in Kendari City, Southeast Sulawesi. The edible film materials used in this study include ĸ-CAR sourced from PT. Kappa Carrageenan Nusantara in Pasuruan, East Java, Indonesia; SOR powder obtained from Ueno Fine Chemicals Industry Ltd., in Thailand; and GLY from PT. Sinar Mas Agro Resources and Technology Tbk. in Jakarta, Indonesia. Effective microorganism 4 (EM4) composting solution was produced by PT. Songgolangit Persada, Jakarta, Indonesia. The chemicals used in this study include hydrochloric acid (HCl) and sodium hydroxide (NaOH) obtained from Merck in Germany and distilled water sourced from Waterone, PT. Jayamas Medica Industri, Indonesia.

### 2.2. Methods

#### 2.2.1. Gelatin Preparation

**Figure 1:**
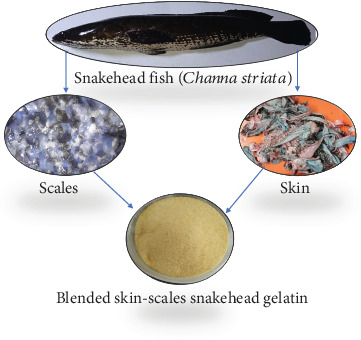
Snakehead fish and snakehead gelatin sources.

The procedure of extracting gelatin from snakehead fish has been investigated by Rosmawati et al. [13] in prior studies. The scales and skin of snakehead fish were cleansed of diverse contaminants using running water. The scales underwent demineralization by immersion in a 3% HCl solution 1:5 (*w*/*v*) for 24 h. To release fat, the skin is immersed in a solution of 10% butyl alcohol 1:4 (*v*/*v*) for 24 h. Each specimen was thoroughly rinsed with running water on three separate occasions to eliminate any remaining traces of the soaking solution. To eliminate any remaining traces of the soaking solution, each sample was subjected to a thorough rinsing process with running water, repeated three times. The skin or scales were immersed in a 0.5 M NaOH 1:5 (*w*/*v*). This process lasts 3 h, with the solution replaced every hour. Periodic stirring facilitates the soaking solution's release and dissolution of noncollagen components. The scales or skin are rinsed three times with running water and then drained. In the next phase, the scales or skin samples were soaked in a solution of 0.05 M acetic acid 1:5 (*w*/*v*) for 6 h. This step is aimed at facilitating the cleavage of the peptide bond. Subsequently, the samples were rinsed thoroughly using running water, which was repeated three times. In the end phase, the scales and skin were rinsed with distilled water, and both were mixed and subjected to extraction using distilled water 1:5 (*w*/*v*) for 15 h in a water bath. The gelatin solution that had been prepared was subjected to a drying process in an oven set at a temperature of 60°C for 48 h. The raw material of SG is shown in Figure 1.

#### 2.2.2. Edible Film Preparation

**Figure 2:**
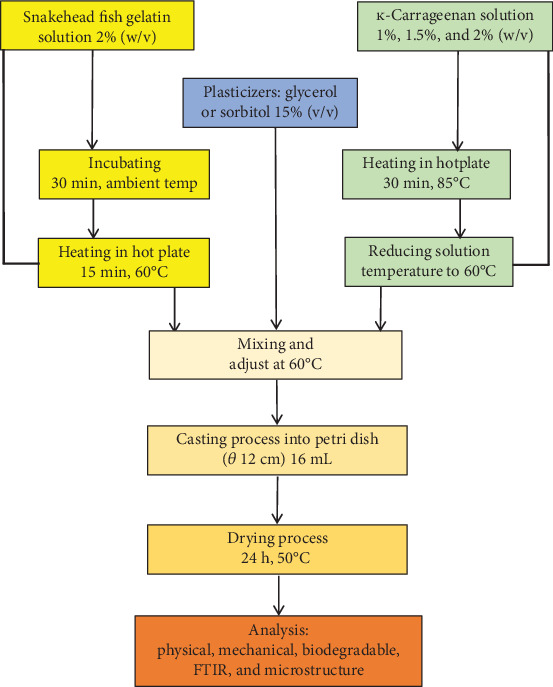
Flow chart edible film preparation.

A solution of SG at a concentration of 2% (*w*/*v*) was mixed with ĸ-CAR at concentrations of 1%, 1.5%, and 2% (*w*/*v*). The plasticizer used was either SOR or GLY, each at a concentration of 15% of the total material (*v*/*v*). The treatment formulations are displayed in Table 1. The fish gelatin was dissolved in aqueous solution and incubated for 30 min at ambient temperature. The SG solution was afterwards heated on a hot plate with continuous stirring, reaching a temperature of 60°C for 15 min. The solution of ĸ-CAR was dissolved in aqueous media and heated at 85°C for 30 min. The solution's temperature was reduced to 60°C before SG and SOR or GLY solutions were combined. A 16-mL film solution was introduced into a petri dish with a diameter of 12 cm and afterwards subjected to a drying process lasting 24 h at a temperature of 50°C. The dry film was stored in a desiccator at a controlled temperature of 25°C ± 2°C and relative humidity of 55% ± 5% before analysis. The edible film production flow is presented in Figure 2.

#### 2.2.3. Thickness and Color

Micrometers (MDC-25M, Mitutoyo, MFG, Japan) were used to measure the thickness of the film with an accuracy of 0.01 mm. Measurements were taken in every sample at five separate locations to determine the average thickness and replicated in five different samples. Using a colorimeter (Color Reader Flex E2, Minolta, Japan), the brightness (*L*^∗^), redness (*a*^∗^), and yellowness (*b*^∗^) of the film are measured and compared to the standard color. The *L*^∗^, *a*^∗^, and *b*^∗^ calibration values were 92.538, −0.866, and 2.655, respectively. Equation (1) calculates the film's overall color difference (Δ*E*):

(1) $$\begin{array}{c} \Delta E=\sqrt{{\left({\Delta L}^{*}\right)}^{2}+{\left({\Delta a}^{*}\right)}^{2}+{\left({\Delta b}^{*}\right)}^{2}} \end{array}$$

#### 2.2.4. Mechanical Characteristics

Referring to Maruddin et al. [34], digital gauge HF 500 was used to measure TS (MPa) and elongation at break (EAB) (%). An area of 15 mm was placed on either side of the length of an edible film that was 8 × 3 cm in size and attached horizontally to the hook. After sampling, the TS value was read (Equation (2)), and the EAB value was calculated using an analysis of the deformation–force curve at the breakpoint (Equation (3)):

(2) $$\begin{array}{c} \text{TS }\left(\text{MPa}\right)=\frac{\text{maximum force }\left(\text{N}\right)}{\text{film thickness}\times \text{width }\left({\text{cm}}^{2}\right)} \end{array}$$

(3) $$\begin{array}{c} \text{EAB }\left(\%\right)=\left(\frac{\text{film elongation}}{\text{initial length}}\right)\times 100\% \end{array}$$

#### 2.2.5. Water Vapor Transmission Rate (WVTR)

The modified ASTM E 96-95 [35] standard method was used to assess if water vapor can permeate the film at a specific temperature and relative humidity. Ten hours was spent heating the glass WVTR to 60°C. The glass was chilled, weighed empty, filled with 3 g of silica gel, and weighed again. A 28-mm-diameter circle was cut out of the film. Reweighing was done once the cut film was set down on the glass WVTR surface. The sample glass was kept in a controlled desiccator at a temperature of 25°C ± 2°C and a relative humidity of 55% ± 2%. The glass was taken out of the desiccator and weighed every hour. There were eight iterations of this technique (1–8 h). Equation (4) was used to express the value in gram per square millimeter per hour units:

(4) $$\begin{array}{c} \text{WVTR}=\frac{n}{t\times A} \end{array}$$

where *n* = weight change (g), *t* = time (hour), and *A* = the surface film area (mm).

#### 2.2.6. Swelling Index (SI)

In evaluating the SI, the modified techniques of Hashemi and Mousavi Khaneghah [36] and Moradi et al. [37] were used. The films, measuring 20 × 20 mm, underwent a desiccation process lasting 24 h at a temperature of 25°C ± 2°C and a humidity level of 55% ± 5% before being subjected to the test. After weighing the sample to ascertain its initial weight (*W*_0_), it was immersed in a petri dish containing 30 mL of distilled water. The sample was allowed to stand at room temperature for 2 min. After that, it was dried using filter paper and weighed to find its final weight (*W*_*f*_). Equation (5) yields the SI:

(5) $$\begin{array}{c} \text{SI}\left(\%\right)=\left[W_{f}-W_{0}/W_{0}\right]\times 100\% \end{array}$$

#### 2.2.7. Solubility Film (SF)

Film samples with dimensions of 20 × 20 mm were examined by Khazaei et al. [38]. The piece sample was dried for 24 h at 105°C in the oven, and then, it was weighed to determine its initial weight (*w*_*i*_). The weighted sample was then kept at room temperature for 24 h in a petri dish with 30 mL of distilled water. To be able to determine the final dry weight (*w*_*f*_), the remaining film segments are filtered using a filter paper and dried once again, just like they were initially. Equation (6) reports the percentage of solubility of the film as the difference between the initial dry weight (*w*_*f*_) and the end insoluble dry weight (*w*_*i*_):

(6) $$\begin{array}{c} \text{SF }\left(\%\right)=\frac{W_{i-}\;W_{f}}{W_{i}}\times 100\% \end{array}$$

#### 2.2.8. Moisture Content (MC)

The film's MC was assessed using the methodology outlined by Uranga et al. [39]. The initial weight (*w*_*i*_) of films measuring 20 × 20 mm was determined, followed by their drying in an oven at 105°C for 24 h. The film was allowed to equilibrate to ambient temperature before being reweighed (*w*_*f*_). The determination of water content was conducted using Equation (7):

(7) $$\begin{array}{c} \text{MC }\left(\%\right)=\frac{W_{i}-W_{f}}{W_{i}}\times 100\% \end{array}$$

#### 2.2.9. Contact Angle (CA) (*θ*)

Determination of the hydrophobicity of the film is achieved through measuring the CA (°) indicated by the film surface. The CA test was determined based on the sessile drop methodology referring to the method of Pouralkhas et al. [3], which has been modified. A 20 × 20 mm film was placed on a glass slide, and 5 *μ*L of distilled water was dropped on its surface. The position of the water droplets on the film was captured with a Nikon 3200 35-mm fixed lens camera at time intervals of 0, 15, 20, 45, and 60 s. The experimental protocol involved replicating each treatment five times. The level of contact is determined using the CorelDraw program.

#### 2.2.10. Biodegradation Test

The determination of the film's weight loss (WL) was conducted using the modified methodology proposed by Nouraddini, Esmaiili, and Mohtarami [40] and Susmitha et al. [41]. Film samples with dimensions of 20 × 20 mm and their initial weight were measured as *W*_0_. The composting procedure involves the insertion of samples at a depth of 3 cm into a container filled with soil. The soil was rinsed with a solution of diluted liquid EM4 containing *Lactobacillus* sp., *Saccharomyces* sp., and cellulosic and ligninolytic bacteria. Before utilization, the EM4 solution was combined with molasses and distilled water, resulting in dissolution. The molasses was prepared by mixing 10 g of crushed brown sugar–type palm (*Arenga pinnata*) with 10 mL of distilled water. The EM4 solution was created by combining 20 mL of EM4, 20 mL of molasses, and 960 mL of distilled water. The process of composting soil involves the maintenance of moisture levels by regular watering. Samples were weighed at regular intervals every 7 days (*W*_*dn*_), following a thorough cleaning process involving careful washing and drying with filter paper (Equation (8)):

(8) $$\begin{array}{c} \text{WL }\left(\%\right)=\frac{W_{0}-W_{dn}}{W_{0}}\times 100\% \end{array}$$

where (*W*_0_) is the initial weight and (*W*_*dn*_) is the weight after degradation on the *n*-day, *n* = 0 , 7, 14, 21, and 28 days.

#### 2.2.11. Fourier Transform Infrared (FTIR)

The FTIR spectrophotometer model Prestige-21, FTIR-8400, manufactured by Shimadzu in Japan, was employed to analyze the edible film samples. The analysis was conducted using the methodology described by Rosmawati et al. [13]. The film's functional group analysis result was obtained by preparing each sample weighing 2 mg and ensuring consistent MC after storing it in a 50°C oven. The samples were coated with flour and augmented with 100 mg of potassium bromide (KBr) before being inserted into a crystal spectrophotometer. The spectrum was measured within the wavenumber range of 4000–650 cm^−1^. FTIR analysis was employed to identify the functional groups and chemical interactions between biomaterials and plasticizers.

#### 2.2.12. Scanning Electron Microscopy (SEM)

Surface micrographs of six films were observed using a SEM (Hitachi SU–3500, Japan). Edible films measuring 20 × 15 mm were coated with 20 mA Au as a conductor that would reflect electrons to the microscope detector. The films were inserted into a specimen chamber set to a magnification of 7.1–7.6 mm with a speed of 3.00 kV.

### 2.3. Statistical Analysis

The analysis of all tests was conducted in five duplicates. The samples underwent variance analysis (ANOVA) using SPSS 16.0.0 software (SPSS Inc., Chicago, Illinois, United States). The data was presented using average values and corresponding standard deviations (SDs). The statistical significance (*p* < 0.05) of the differences between treatments was consistently observed per the Duncan test.

## 3. Results and Discussion

### 3.1. The General Appearance of Edible Film

Edible film composite–based SG with ĸ-CAR in various concentrations and the addition of SOR or GLY concentrations have been successfully prepared. The appearance of films between the C1G2Sor, C1.5G2Sor, C2G2Sor, C1G2Gly, C1.5G2Gly, and C2G2Gly treatments was relatively different due to the various ĸ-CAR concentrations and plasticizers used. Films using plasticizer SOR appear more transparent but less flexible and tend to wrinkle when removed from the plate. This is caused by the difficulty of separating the film from the plate, such as palm starch film to which 15% SOR was added [42], which causes the film to wrinkle. These wrinkles can cause brittleness due to tensile stress when peeled. On the other hand, the use of GLY as a plasticizer produces a film that is more flexible and peels easily but feels a little wet and sticky and tends to be less transparent compared to films added with SOR [43, 44]. The two additional ingredients (SOR and GLY) are types of polyol plasticizers with low molecular weight. These plasticizers can damage hydrogen bonds in the intermolecular/intramolecular polymers that make up the film [43, 45]. The addition of plasticizers can reduce the affinity between molecules, forming new hydrogen bonds with the plasticizer, thereby increasing flexibility and preventing damage to the film [45]. Still, according to Murrieta-Martínez et al. [46], long-term storage will facilitate the diffusion of molecules to the surface, thus making the structure brittle.

### 3.2. Thickness and Measurement of Color

The measurement of film thickness is a crucial characteristic that significantly correlates with many physical and mechanical properties, including TS and elongation. Therefore, it is imperative to allocate due attention to this aspect. The experimental that received a combination treatment of SG, ĸ-CAR, and plasticizers (SOR and GLY) exhibited statistically significant variations (*p* < 0.05) in film thickness (Table 2). The level of film thickness produced was influenced by the combination of SG/ĸ-CAR at varying concentrations. Specifically, an increase in the concentration of ĸ-CAR leads to a corresponding rise in film thickness. This study resembles the starch/ɪ-CAR composition conducted by Thakur et al. [29]. According to Ghasemlou, Khodaiyan, and Oromiehie [43], there appears to be a correlation between the biopolymer composition, the concentration of film constituents, and the resulting film thickness. The film exhibits a substantially higher thickness when SOR is included, compared to GLY. In general, the C2G2Sor treatment revealed a greater film thickness magnitude than the remaining treatments, namely, 0.046 ± 0.005 mm. This thickness was smaller than the standard set by Japanese Industrial Standards (JIS), namely, 0.25 mm [47]. However, Pavlath and Orts [48] stated that a film thickness of less than 0.060 mm would have a reduced effect on water vapor transfer compared to a greater thickness.

The utilization of various plasticizers can influence the presence of disparities in the thickness of a particular variety of films. The film exhibits a slightly increased thickness when SOR was included, as observed by the findings of Sanyang et al. [42]. According to Ghasemlou, Khodaiyan, and Oromiehie [43], the rapid and efficient penetration of polymer networks by SOR, compared to GLY, enables it to create thin films effectively. In comparison to GLY, SOR possesses a higher number of hydroxyl groups. Consequently, the presence of these hydroxyl groups enhances the affinity of SOR towards the gelatin protein network, leading to a relatively thicker layer [32]. When combined with plasticizers, biomaterials' molecular conformation has been observed to influence the measured values. This phenomenon has been demonstrated in studies involving corn starch and gelatin with the addition of mango, mango pure or pure mango peel, and pineapple pomace [41].

The film's color is essential as it directly impacts the product's visual aesthetics and consumer perception. The results of the influence of the composite treatment, comprising a biomaterial and a plasticizer, on the luminosity of the film are presented in Table 2. The concentration of ĸ-CAR did not affect the brightness value (*L*^∗^) of the film (*p* > 0.05); however, the addition of different plasticizers showed a change in the *L* value. The brightness of the film containing GLY was higher than SOR. The brightness (*L*^∗^) values, encompassing a range of 72.78–85.98. Our temporary suspicion is that the difference in brightness of this film is also likely to be intervened by the presence of SG. Visually, the color of the two plasticizers appears clear, while the *L*^∗^ value of SG was 61 [13]. Compounding biomaterials with plasticizers may have disrupted the bond between each other, resulting in different film colors. No statistically significant variation (*p* > 0.05) was detected in redness (*a*^∗^) across all treatments. However, there were differences in the yellowness value (*b*^∗^). The redness value of the film ranges from −1.42 to 1.28. Different ĸ-CAR concentrations did not show differences in *Δ*E values (*p* > 0.05), but the addition of SOR resulted in higher *Δ*E values than those treated with GLY (*p* < 0.05). According to Musso, Salgado, and Mauri [49], color differences in edible films can be attributed to the specific biomaterials used and the type of plasticizer added.

### 3.3. Tensile Properties

TS between treatments showed significant differences (*p* < 0.05) (Table 3). The higher the concentration of ĸ-CAR, the more the TS tends to increase. The highest TS was in the C1.5G2Sor and C2G2Sor films, and the lowest was in the C1G2Gly films. Among the films treated with SOR or GLY, films from the SG/ĸ-CAR/SOR combination had higher values, ranging from 0.63 to 2.05 MPa, than those from the SG/ĸ-CAR/GLY combination, which was 0.18–1.03 MPa. The composition of the film contributes to the TS value, and according to Hammam [6], gelatin protein tends to have low tensile capacity compared to other polymers. However, the combination of gelatin with other polymers can increase the TS of the film [3, 50, 51]. As a result, SG/ĸ-CAR/SOR treatment showed an increase in film TS, although, for some films, the value may differ depending on the type of biopolymer used [5]. According to JIS, the standard value for TS was more than 0.392 MPa [47]. As shown in Table 3, the TS values of the C1G2Gly and C1.5G2Gly treatments do not fulfill JIS criteria, implying that SG and CAR combination films weighing 1–1.5 g and containing 15% GLY were not approved. On the other hand, the role of plasticizer has a significant effect on the TS value, as Pouralkhas et al. [3] found that films with GLY plasticizer had lower values than SOR. It may have something to do with SOR's more considerable molecular weight and weaker hygroscopicity, causing the formation of strong bonds between intra- and molecular interactions of the film constituent materials [52].

In addition to TS, EAB encompasses significant mechanical properties of the film. The treatment of the SG/ĸ-CAR/plasticizer combination influences the percentage of EAB (*p* < 0.05). However, as depicted in Table 3, it was evident that the EAB percentage for the treatments C1G2Sor, C1.5G2Sor, C2G2Sor, and C1G2Gly was generally similar, ranging from 22.40% to 25.20%. Notably, these values were lower than the EAB percentage for the treatments C1.5G2Gly and C2G2Gly, which vary from 34.4% to 35.00%. However, the EAB percentage range was still considered good according to the JIS criteria: no less than 10% as very bad criteria and no more than 50% as perfect [47].

The composition of biopolymers utilized in the production of edible films has the potential to influence the EAB. According to a study conducted by Nurdiani et al. [53], it was found that the combination of gelatin and fucoidan leads to an increase in EAB as the concentration of fucoidan increases. The combination of SG/ĸ-CAR/SOR did not significantly impact film elongation. However, the variety of SG/ĸ-CAR/GLY exhibited an enhanced capacity of the film to stretch until it reached the point of breakage. These conditions resembled the findings of Bakry, Isa, and Sarbon [32] regarding incorporating SOR or GLY into the gelatin/carboxymethyl starch/chitosan composite film. The ratio of protein to polysaccharide can impact the film's mechanical properties [3]. According to Pranoto, Lee, and Park [54], the ideal proportion of gelatin and polysaccharide for achieving an improved combination was 2%. Additionally, Fu et al. [33] found that GLY was more effective than SOR in enhancing EAB. Hence, this study has to offer novel perspectives on using SG as an innovative biomaterial in the fabrication of films for wider-related purposes.

### 3.4. WVTR

The findings from the analysis of the WVTR of the film, as presented in Table 3, indicate a statistically significant variation (*p* < 0.05) among the different treatments. The WVTR exhibits a percentage range of 0.17–0.28 g/mm^2^/h. The JIS limits the WVTR value to 7 g/m^2^/day (≈2.92 × 10^−7^ g/mm^2^/h), which is lower than the observed amount. This effect was associated with the type of biomaterial employed. ĸ-CAR was a hydrophilic biomaterial [21] with a more hydrated structure and moister surface, allowing water molecules to migrate more easily.

The combination of SG/ĸ-CAR/SOR demonstrates a decrease in WVTR as the concentration of ĸ-CAR increases. According to Govindaswamy et al. [55], it has been shown that the utilization of SOR has the potential to decrease the value of WVTR. However, in the context of the SG/ĸ-CAR/GLY treatment, there was a tendency for the WVTR to increase. The prominence of both plasticizers significantly impacts the percentage of WVTR. The distinct molecular weights of plasticizers influence the hydrogen bonding and inter-/intramolecular interactions in SG/ĸ-CAR.

Furthermore, variations in the WVTR were seen when different concentrations of SG/ĸ-CAR combinations were utilized. The WVTR value of the film was determined by the intermolecular interactions among its constituent molecules [33]. Additionally, the hydrophilic qualities of the materials employed in the film contribute to its reduced ability to allow water vapor transmission [56]. High WVTR values were less appropriate for packaging high-water-content food products but were recommended for drier ones. This information was important to determine the film's purpose for a specific packaging. Thus, the incorporation of SG/ĸ-CAR with hydrophobic biomaterials such as lipids, essential oils, or phenolic acids [2, 21, 56] requires further study.

### 3.5. SI

The findings of the study revealed a significant difference (*p* < 0.05) in the SI across the various treatments (Table 4). The utilization of SG/ĸ-CAR at varying concentrations implies a discernible variation in the film's susceptibility to undergo swelling. There is a direct relationship between the concentration of ĸ-CAR and the SI. The inclusion of SOR or GLY in the SG/ĸ-CAR mixture yielded contrasting outcomes. Specifically, adding SOR resulted in substantially higher values (ranging from 49.77% to 68.87%), while including GLY led to considerably lower values (ranging from 35.51% to 53.63%). The SI of a film can be affected by the incorporation of biopolymers into formulations [41], as well as the occurrence of strong intermolecular interactions, which can reduce the integration of the film matrix and potentially result in film rupture [36]. The film's high SI can be ascribed to its capacity to absorb and hold moisture effectively. The influences of biopolymer selection on the hydrophilicity of ĸ-CAR have been observed to substantially impact the properties of this phenomenon [5, 57]. Additionally, the relative balance between SG proteins' hydrophilic and hydrophobic properties has been identified as a contributing factor [5, 12]. Understanding the SI value can aid in determining the appropriate application of an edible film based on the specific attributes of the food intended for packaging.

### 3.6. SF

The primary purpose of the film is to provide a protective barrier for food products against water, thus emphasizing the crucial role of the film's solubility. The solubility of SG-based edible films, treated with different concentrations of ĸ-CAR and plasticizers, demonstrated significant variations in different treatments (*p* < 0.05). The solubility of C1.5G2Sor and C2G2Sor films was equivalent, as was observed between C1G2Sor, C1G2Gly, C1.5G2Gly, and C2G2Gly, correspondingly. Including SOR at a concentration of 1%–2% in the ĸ-CAR solution led to a notable reduction in the proportion of SF. However, the presence of GLY at different concentrations in the ĸ-CAR solution did not exhibit any discernible variation among the treatments. The solubility value showed minimal variation compared to the findings reported by Ghaderi et al. [58] in their study on the composite film of chitosan/polyvinyl alcohol/gelatin of cold water fish incorporating GLY. The film exhibits contrasting solubility characteristics between SOR and GLY, as observed in the study conducted by Ballesteros-Mártinez, Pérez-Cervera, and Andrade-Pizarro [44] about sweet potato starch. Plasticizers have been seen to influence the solubility characteristics of films. Paudel, Regmi, and Janaswamy [59] have reported that films containing GLY exhibit comparatively more excellent solubility than those containing SOR. The study mentioned above by Murrieta-Martínez et al. [46] elucidates an asymmetry in the molecular structure resulting from insufficient interaction between the protein and the plasticizer. Consequently, this interaction impedes the production of well-structured tissue incorporating gelatin. Accordingly, the SF content was efficacious in food-oriented product applications that exhibit rapid dissolution in the oral cavity [44].

### 3.7. MC

Table 4 shows that significant changes in the MC of SG/ĸ-CAR films at various concentrations were observed (*p* < 0.05). As the concentration of ĸ-CAR increases, a corresponding drop in MC occurs when SOR and GLY were introduced. Nevertheless, it was commonly seen that films incorporating SOR exhibit comparatively lower MC percentages, ranging from 15.74% to 20.29%, in contrast to films containing GLY, which display MC percentages ranging from 21.66% to 29.31%. The observed effect resembles the one seen in kefiran–GLY films, where MC was higher compared to kefiran–SOR films within the concentration range of 15%–35% [43]. There were no standard rules for the MC of the film. Ahmed and Janaswamy [60] asserted that MC was related to the amount of water contained in a material and was positively correlated with water absorption, water solubility, elongation percentage, and biodegradability but negatively correlated with TS. MC was critical for adapting packaging requirements to product stability. The type of raw material and manufacturing processes had a great influence on film MC variations. The observed decrease in MC in the film SG/ĸ-CAR/SOR was believed to be attributed to the robust connections between SOR and polymer chains within the molecular structure.

Consequently, SOR exhibits little interaction with MC, as Hazrol et al. [61] suggested. On the other hand, in the context of GLY, it was observed that the hydroxyl group exhibits a significant attraction towards water molecules, increasing the MC. This increased MC enables the matrix to retain water and establish hydrogen bonding interactions [62]. The higher the MC, the more the space that can be filled by water molecules, which leads to the number of crosslinks formed from the performance of biomaterials and plasticizers. Strong crosslinks can slow down the trapping of water molecules by the film layer [60]. In the context of water absorption, Fick's law explains how substances diffuse through materials. However, this phenomenon was largely determined by relative humidity and temperature [48].

### 3.8. CA (*θ*)

**Figure 3:**
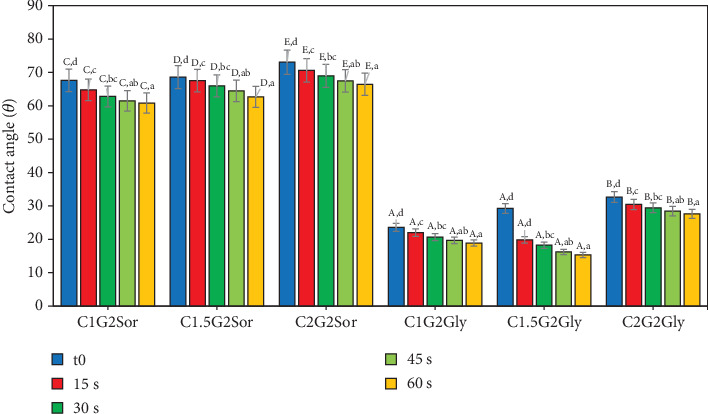
The rate of decrease in the contact angle of edible snakehead gelatin–based films with ĸ-carrageenan of different concentrations plasticized with sorbitol or glycerol. A, B, C, D, E and a, b, c, d, e: values with the same superscript are not significantly different (*p* > 0.05).

The hydrophobicity level of an edible film can be assessed by quantifying the CA, which indicates the film's surface tension [63]. The contact angle of the film, denoted as CA, was measured for a time interval of 0 to 60 s, as presented in Table 4, which displays the average CA. The hydrophobicity properties of the film illustrated as a rate function as depicted in Figure 3 revealed a significant decrease in the water repellency of the films as time progressed. The experimental results showed that the film's CA changed significantly across different treatments statistically considerably (*p* < 0.05). The degrees of CA for C1G2Gly and C1.5G2Gly films exhibit the lowest values in comparison to C2G2Gly; however, the C1G2Sor, C1.5G2Sor, and C2G2Sor films demonstrate very similar CA degrees. SG and ĸ-CAR were hydrocolloids known for their favourable hygroscopic characteristics [7]. Khazaei et al. [38] found similarities in hygroscopic characteristics between SOR and GLY.

Additionally, the addition of one of them to the SG/ĸ-CAR formulation was observed to result in a decrease in film hydrophobicity. The film's hydrophobic nature can be elucidated through the measurement of the CA, as discussed by Ahmed and Janaswamy [60]. Furthermore, previous research conducted by Farhan and Hani [1] has demonstrated that SOR exhibits higher hydrophobicity than GLY. This phenomenon appears to be associated with the hindrance of polar groups in their ability to authenticate with the surface, as discussed by Zuraini et al. [64]. Valencia et al. [63] conducted a study on oil-free edible film and made observations regarding the decline in the wetness retention capacity of the film's surface layer.

### 3.9. Biodegradability Film

**Figure 4:**
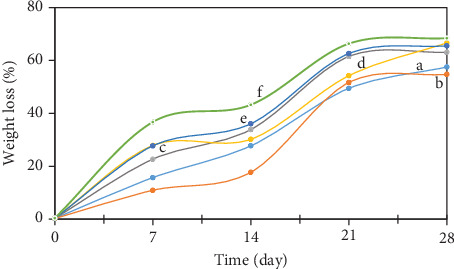
Weight loss of edible film–based gelatin of snakehead fish with ĸ-carrageenan of different concentrations plasticized with sorbitol or glycerol (a = C1G2Sor; b = C1.5G2Sor; c = C2G2Sor; d = C1G2Gly; e = C1.5G2Gly; and f = C2G2Gly).

The edible films, based on SG/ĸ-CAR, with varying concentrations and plasticized with SOR or GLY, exhibited a significant rate of WL of the film (*p* < 0.05), as depicted in Figure 4. The edible film appeared to initiate process WL on the seventh day across all treatment groups. Until Day 28, the recorded observations indicate that the weight reduction of the treatment film had attained approximately 54%–63% in films that were plasticized with SOR. In contrast, films plasticized with GLY showed weight loss ranging from 65% to 68%. The decomposition capacity of the films appeared to be influenced by the plasticizer and the material properties, thus affecting the biodegradation behavior of the films [59]. GLY exhibited higher hydrophilicity than SOR [1]. The high water absorption and water vapor permeability of films plasticized with GLY resulted in slightly faster degradation than films with SOR [59]. This is evident from the disintegration rate observed in films with GLY, which occurred slightly faster than in films with SOR.

Another observed phenomenon can be attributed to the higher molecular weight of SOR, which results in a lower solubility rate than GLY, which in turn affects a slightly slower biodegradation rate [32]. The capacity of SG and ĸ-CAR to interact easily with water and microbes led to their classification as biomaterials. The degrading process of the edible film entails the intentional application of several microorganisms, such as *Lactobacillus* sp., *Saccharomyces* sp., cellulosic bacteria, and ligninolytic bacteria to the soil media. Microorganisms, including fungi and bacteria, contribute to film degradation [65] by facilitating oxidation, hydrolysis, and enzymatic digestion, efficiently executed by microbial activity. The film disintegration process of the therapy was well performed, as evidenced by the progressive increase in film WL seen throughout the treatment.

### 3.10. Functional Group Identification

**Figure 5:**
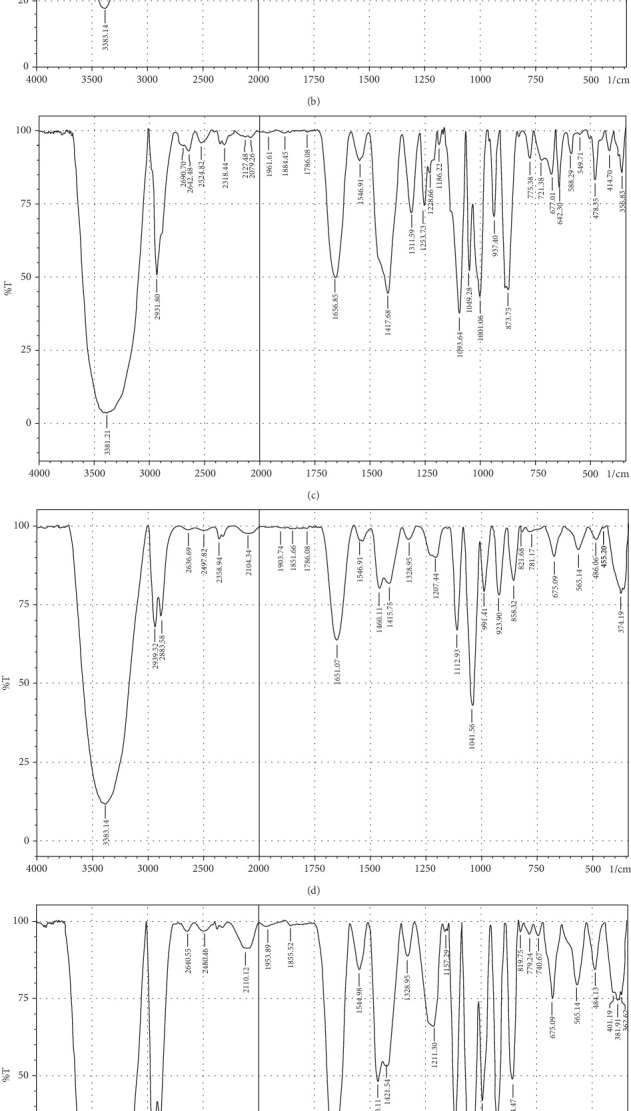
Comparison of the Fourier transform infrared (FTIR) spectra of edible film–based snakehead gelatin with ĸ-carrageenan of different concentrations plasticized with sorbitol or glycerol. (a) C1G2Sor; (b) C1.5G2Sor; (c) C2G2Sor; (d) C1G2Gly; (e) C1.5G2Gly; (f) C2G2Gly.

The purpose of employing an FTIR spectrophotometer in film characterization was to assess the interaction between SG and ĸ-CAR by utilising infrared waves inside the film matrix [55, 66]. The spectrum of the complete treatment is depicted in Figure 5. At the same time, Table 5 provides a compilation of the characteristic wavenumbers utilized for identifying and characterizing interactions among the constituent biomaterials comprising the edible film. Almost all samples showed hydroxyl bands at wave numbers 3381.21–3446.79 cm^−1^ (Amide A). Pranoto, Marseno, and Rahmawati [66] revealed the existence of combination films composed of tilapia fish gelatin, ĸ-CAR, and gellan, which exhibited characteristic wavenumbers of 3315.04 and 3318.90 cm^−1^. Hazrol et al. [61] state that the gelatin spectrum extends from 3000 to 3600 cm^−1^, encompassing all films irrespective of the plasticizer employed. According to Susmitha et al. [41], it may be inferred that all films possess a uniform functional group structure characterized by the presence of O–H groups. The presence of wavenumbers within a specific range suggests stretching inside the Amide A band. This implies the presence of numerous unbound hydroxyl groups within the structure of the SG/ĸ-CAR film, leading to the elongated vibration of these hydroxyl groups [52].

The peak corresponding to Amide B in SG/ĸ-CAR-based films was observed at wavenumbers ranging from 2931.8 to 2939.52 cm^−1^ (asymmetric) throughout the film, due to C-H stretching vibrations [41, 67]. The peak at wavenumber 2883.58 cm^−1^ (symmetric) was observed in GLY-containing films and was absent in SOR films. It is suspected that there was a spectrum shift in SOR-containing films, thus successfully forming new bonds of gelatin–CAR composites. The ĸ-CAR treatment and inclusion of plasticizers were associated with the formation of gelatin-CAR composites, which was in line with the findings reported by Pouralkhas et al. [3] in their study on gelatin/fucoidan films derived from *Sargassum tenerrimum*. The spectral range for films treated with SOR addition was between 2642.7 and 2644.41 cm^−1^, while in GLY, it varied from 2636.69 to 2659.84 cm^−1^. There appears to be a change in the wavenumber of the films when using GLY instead of SOR. The observed phenomena could be attributed to the hydrophilic characteristics of GLY and SOR, as discussed by Farhan and Hani [1]. There is a contribution of the variations in the amount of ĸ-CAR used. The observed wavenumber shift is attributed to the absorption occurring at the C-H bonds. Although SOR and GLY have different properties, both show wavenumbers in the same range.

The treated film's Amide I band showed its highest intensity in the spectral region of 1643.35–1656.85 cm^−1^. According to the previously indicated assertion, the SG/ĸ-CAR combination film has a notable interconnectivity in its film structure, which the -CONH_2_ group explains. This result aligns with the hypothesis proposed by Hidayati et al. [65]. Kowalczyk et al. [68] state that the stretching vibrations of the carbonyl functional group (C=O) were represented by the spectral peaks observed in the 1633–1646 cm^−1^ region. The wavenumber range in the spectrum of the test film, defined explicitly as Amide II, was between 1544.98 and 1553.7 cm^−1^. This spectral region is distinguished by the presence of vibrational bending originating from the N-H group and the stretching vibration of the C-N group, as reported by Tongnuanchan, Benjakul, and Prodpran [69]. The Amide III film peaks within the spectral region of 1207.44–1251.8 cm^−1^. The observed wavenumber arises from the vibrational modes exhibited by the C-N and N-H groups inside the bonded amide and the vibrational modes originating from the CH_2_ glycine groups in gelatin. The functional group analysis reveals no evidence of new compounds being formed involving SG/ĸ-CAR/plasticizer. However, it can be stated that the edible film tends to exhibit different characteristics due to the addition of different plasticizers based on the visible wavenumbers, which was indicated by a shift in the wave peak in the form of stretching or bending.

### 3.11. SEM

**Figure 6:**
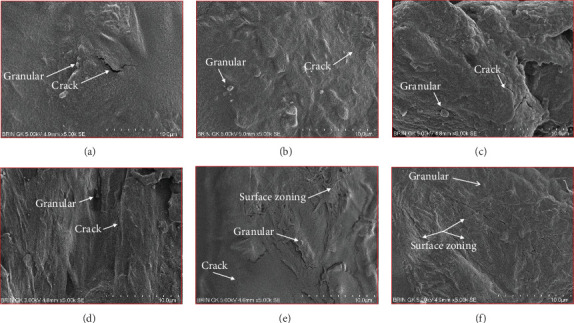
Scanning electron microscopy (SEM) image of edible film based on snakehead gelatin with different concentrations of ĸ-carrageenan plasticized with sorbitol or glycerol in the size range of 10 *μ*m. (a) C1G2Sor; (b) C1.5G2Sor; (c) C2G2Sor; (d) C1G2Gly; (e) C1.5G2Gly; (f) C2G2Gly.

The microstructure of edible films was observed to explore their structure and morphology, including film homogeneity, cracks, layer structure, pores, surface, smoothness, and thickness, which directly affect gas permeability [55]. The SG/ĸ-CAR combination film plasticized with SOR showed a rougher surface, cracks, and granules suspected to be ĸ-CAR that did not fully dissolve together with gelatin. The higher the concentration of ĸ-CAR, the denser, thicker, and wavy but more cohesive the film surface with the appearance of finer cracks (Figures 6(a), 6(b), and 6(c)). Bakry, Isa, and Sarbon [32] explain that SOR can result in a higher affinity of protein network to bind through hydroxyl groups, resulting in a denser film with thicker and stronger film properties. These findings were confirmed in the film thickness in Table 2. A smoother surface structure with fainter cracks than the SOR-plasticized film characterized the film associated with GLY, as shown in Figure 6(a) versus Figure 6(d). The surface of C1.5G2Gly and C2G2Gly films (Figures 6(e) and 6(f)) formed a zoning structure as an effect of increasing the concentration of ĸ-CAR. The relationship between ĸ-CAR and SG facilitated by GLY can disrupt the affinity, thus displaying irregular zones on the SG/ĸ-CAR/GLY film. This event was due to the formation of preferential channels during the drying process [55]. Observations by Kowalczyk et al. [70] on the combination of gelatin and carboxymethyl cellulose and GLY as plasticizers showed different surface structures from each other, which was assumed to be the effect of the addition of other substances in the composition. When associated with the results of the functional group analysis, the film added with SOR was more compact as an indication of the bonding between SG and ĸ-CAR into a composite. This was in line with Farhan and Hani [1], who stated that adding SOR can cause structural changes in the film. However, the advantage of the film plasticized with GLY was that it is more flexible than the SOR-plasticized film. The flexibility of the film is related to the hygroscopic properties and the number of OH molecules [1], and FTIR has confirmed this.

## 4. Conclusion

The combination of SG with ĸ-CAR in different concentrations, which was plasticized using SOR or GLY, had relatively different physical, mechanical, and biodegradability characteristics. Films plasticized with GLY demonstrate higher thickness, more excellent EAB, increased WVTR, and enhanced solubility than those plasticized with SOR. Conversely, films plasticized with SOR show a slower decomposition rate and more stable mechanical properties, such as TS and SI, particularly at higher ĸ-CAR concentrations. A key finding is the influence of GLY's higher hydrophilicity over SOR in determining the film's characteristics, including the decreasing water content with increasing ĸ-CAR concentration. Although no new compounds were discovered based on FTIR analysis, a bond was identified between gelatin and ĸ-CAR mediated by SOR. The texture of films plasticized with SOR resulted in a denser and more cohesive surface. The higher the concentration of ĸ-CAR, the thicker and wavier the film surface became, while higher flexibility was found in films using GLY. This study provides crucial insights into the potential use of the unique combination of SG and ĸ-CAR as a base material for developing edible biomaterial films with customizable characteristics for various applications, including eco-friendly packaging. Further research is needed to address the method's limitations, the biomaterial's unstable nature that affects the homogeneity of the edible film layer, and the type and concentration of plasticizer. Challenges to the rigor of the research process instill confidence in the reliability of the findings.

## Figures and Tables

**Table 1:** The formulation of an edible film–based snakehead gelatin with ĸ-carrageenan in varying concentrations then plasticized using sorbitol or glycerol.

**Concentration**	**Sample code**
ĸ-Carrageenan 1% (*b*/*v*) Snakehead gelatin 2% (*w*/*v*) Sorbitol 15% (*v*/*v*)	C1G2Sor

ĸ-Carrageenan 1.5% (*b*/*v*) Snakehead gelatin 2% (*w*/*v*) Sorbitol 15% (*v*/*v*)	C1.5G2Sor

ĸ-Carrageenan 2% (*b*/*v*) Snakehead gelatin 2% (*w*/*v*) Sorbitol 15% (*v*/*v*)	C2G2Sor

ĸ-Carrageenan 1% (*b*/*v*) Snakehead gelatin 2% (*w*/*v*) Glycerol 15% (*v*/*v*)	C1G2Gly

ĸ-Carrageenan 1.5% (*b*/*v*) Snakehead gelatin 2% (*w*/*v*) Glycerol 15% (*v*/*v*)	C1.5G2Gly

ĸ-Carrageenan 2% (*b*/*v*) Snakehead gelatin 2% (*w*/*v*) Glycerol 15% (*v*/*v*)	C2G2Gly

**Table 2:** The thickness and colors of the edible film based on snakehead gelatin with ĸ-carrageenan of different concentrations plasticized with sorbitol or glycerol.

**Parameters**	**C1G2Sor**	**C1.5G2Sor**	**C2G2Sor**	**C1G2Gly**	**C1.5G2Gly**	**C2G2Gly**
Thickness (mm)	0.034 ± 0.005^b^	0.036 ± 0.005^b^	0.046 ± 0.005^c^	0.024 ± 0.005^a^	0.032 ± 0.008^ab^	0.032 ± 0.004^ab^
*L* ^∗^	79.97 ± 1.96^ab^	76.31 ± 1.70^ab^	72.78 ± 1.03^a^	80.60 ± 4.20^bc^	84.99 ± 8.00^c^	85.98 ± 5.09^c^
*a* ^∗^	−0.24 ± 1.00^a^	−0.49 ± 0.48^a^	−0.68 ± 0.77^a^	0.28 ± 0.74^a^	0.04 ± 0.28^a^	−1.42 ± 3.66^a^
*b* ^∗^	−0.32 ± 0.53^a^	−0.97 ± 0.99^a^	−1.13 ± 0.44^a^	−0.56 ± 0.48^a^	−0.58 ± 0.74^a^	−5.15 ± 4.25^b^
Δ*E*	16.63 ± 1.96^bc^	16.25 ± 1.71^bc^	19.77 ± 1.02^c^	12.07 ± 4.19^ab^	7.71 ± 7.92^a^	9.15 ± 1.56^a^

*Note:* Superscripts letters a, b, c, and d show significant differences in the test with ANOVA on the same row. C1G2Sor = combination of ĸ-carrageenan 1%:snakehead gelatin 2%:sorbitol 15%; C1.5G2Sor = combination of ĸ-carrageenan 1.5%:snakehead gelatin combination of ĸ-carrageenan 2%:snakehead gelatin 2%:sorbitol 15%; C1G2Gly = combination of ĸ-carrageenan 1.5%:snakehead gelatin 2%:glycerol 15%; C2G2Gly = combination of ĸ-carrageenan 2%:snakehead gelatin 2%:glycerol 15%; *L*^∗^ = brightness; *a*^∗^ = redness; *b*^∗^ = yellowness; Δ*E* = color difference.

**Table 3:** Tensile strength (TS), elongation at break (EAB), and water vapor transmission rate (WVTR) of the edible film based on snakehead gelatin with ĸ-carrageenan of different concentrations plasticized with sorbitol or glycerol.

**Parameters**	**C1G2Sor**	**C1.5G2Sor**	**C2G2Sor**	**C1G2Gly**	**C1.5G2Gly**	**C2G2Gly**
TS (MPa)	0.63 ± 0.18^bc^	2.02 ± 0.33^d^	2.05 ± 0.56^d^	0.18 ± 0.17^a^	0.35 ± 0.13^ab^	1.03 ± 0.40^c^
EAB (%)	22.60 ± 1.82^a^	22.60 ± 2.30^a^	25.20 ± 1.79^a^	22.40 ± 3.58^a^	34.4 ± 2.30^b^	35.00 ± 2.92^b^
WVTR (g/mm^2^/h)	0.28 ± 0.02^e^	0.19 ± 0.00^b^	0.17 ± 0.00^a^	0.21 ± 0.01^c^	0.23 ± 0.00^d^	0.22 ± 0.00^cd^

*Note:* Superscripts letters a, b, c, d, and e show significant differences in the test with ANOVA on the same row. C1G2Sor = combination of ĸ-carrageenan 1%:snakehead gelatin 2%:sorbitol 15%; C1.5G2Sor = combination of ĸ-carrageenan 1.5%:snakehead gelatin 2%:sorbitol 15%; C2G2Sor = combination of ĸ-carrageenan 2%:snakehead gelatin 2%:sorbitol 15%; C1G2Gly = combination of ĸ-carrageenan 1%:snakehead gelatin 2%:glycerol 15%; C1.5G2Gly = combination of ĸ-carrageenan 1.5%:snakehead gelatin 2%:glycerol 15%; C2G2Gly = combination of ĸ-carrageenan 2%:snakehead gelatin 2%:glycerol 15%.

**Table 4:** Swelling index (SI), solubility film (SF), moisture content (MC), and contact angle (CA) of edible film based on snakehead gelatin with different concentrations of ĸ-carrageenan plasticized with sorbitol or glycerol.

**Parameters**	**C1G2Sor**	**C1.5G2Sor**	**C2G2Sor**	**C1G2Gly**	**C1.5G2Gly**	**C2G2Gly**
SI (%)	49.77 ± 1.47^c^	63.72 ± 1.63^d^	68.87 ± 2.87^e^	35.51 ± 5.14^a^	41.73 ± 3.28^b^	53.63 ± 2.39^c^
SF (%)	71.43 ± 12.39^b^	40.46 ± 10.35^a^	42.67 ± 15.44^a^	62.71 ± 22.49^b^	63.60 ± 7.28^b^	72.96 ± 4.21^b^
MC (%)	20.29 ± 2.43^b^	20.25 ± 0.76^b^	15.74 ± 0.36^a^	29.31 ± 2.95^d^	24.37 ± 3.88^c^	21.66 ± 2.41^bc^
CA (°)	63.50 ± 2.75^c^	65.86 ± 2.37^c^	65.56 ± 3.20^c^	20.97 ± 1.87^a^	19.77 ± 5.58^a^	29.74 ± 1.96^b^

*Note:* Superscripts a, b, c, d, and e show significant differences in the test with ANOVA on the same row. C1G2Sor = combination of ĸ-carrageenan 1%:snakehead gelatin 2%:sorbitol 15%; C1.5G2Sor = combination of ĸ-carrageenan 1.5%:snakehead gelatin 2%:sorbitol 15%; C2G2Sor = combination of ĸ-carrageenan 2%:snakehead gelatin 2%:sorbitol 15%; C1G2Gly = combination of ĸ-carrageenan 1%:snakehead gelatin 2%: glycerol 15%; C1.5G2Gly = combination of ĸ-carrageenan 1.5%:snakehead gelatin 2%:glycerol 15%; C2G2Gly = combination of ĸ-carrageenan 2%:snakehead gelatin 2%:glycerol 15%.

**Table 5:** Assignments of edible films based on snakehead gelatin and ĸ-carrageenan with sorbitol or glycerol using the FTIR spectrum.

**Amide**	**Assignments**	**Wavenumber (cm** ^**−1**^ **)**					
**Absorption peak area (cm** ^**−1**^ **)**		**C1G2Sor**	**C1.5G2Sor**	**C2G2Sor**	**C1G2Gly**	**C1.5G2Gly**	**C2G2Gly**
A (3200–3600)	-OH stretching vibrations	3381.21	3383.14	3381.21	3383.14	3446.79	3381.21
B (3100–2300)	-CH_2_- and > CH stretching and bending vibrations	2931.80	2933.73	2931.8	2939.52	2939.52	2937.59
		—	—	—	2883.58	2883.58	2883.58
	C-H absorption	2642.70	2644.41	2642.48	2636.69	2640.55	2659.84
I (1656–1644)	C=O and C-N stretching	1656.85	1651.07	1647.21	1651.07	1654.92	1643.35
II (1560–1335)	-NH and C-N bending and stretching vibration	1546.91	1548.84	1552.70	1546.91	1544.98	1546.91
III (1240–670)	-OH bending vibrations	1228.66	1251.80	1251.80	1207.44	1211.30	1211.30

## Data Availability

Data will be made available on request.
